# Working memory for time intervals in auditory rhythmic sequences

**DOI:** 10.3389/fpsyg.2014.01329

**Published:** 2014-11-19

**Authors:** Sundeep Teki, Timothy D. Griffiths

**Affiliations:** ^1^Wellcome Trust Centre for Neuroimaging, University College LondonLondon, UK; ^2^Auditory Cognition Group, Institute of Neuroscience, Newcastle UniversityNewcastle upon Tyne, UK; ^3^Laboratoire des Systemes Perceptifs, CNRS UMR 8248, Departement d’Etudes CognitivesEcole Normale Superiere, Paris, France

**Keywords:** working memory, interval timing, time perception, rhythm perception, auditory perception

## Abstract

The brain can hold information about multiple objects in working memory. It is not known, however, whether intervals of time can be stored in memory as distinct items. Here, we developed a novel paradigm to examine temporal memory where listeners were required to reproduce the duration of a single probed interval from a sequence of intervals. We demonstrate that memory performance significantly varies as a function of temporal structure (better memory in regular vs. irregular sequences), interval size (better memory for sub- vs. supra-second intervals), and memory load (poor memory for higher load). In contrast memory performance is invariant to attentional cueing. Our data represent the first systematic investigation of temporal memory in sequences that goes beyond previous work based on single intervals. The results support the emerging hypothesis that time intervals are allocated a working memory resource that varies with the amount of other temporal information in a sequence.

## INTRODUCTION

Perception of time is an essential aspect of human brain function necessary for performing coordinated actions including speech and movement. However, the absence of dedicated neural machinery for temporal processing renders time perception an intriguing problem in neuroscience (James, 1886; Treisman, 1963; Buhusi and Meck, 2005; Karmarkar and Buonomano, 2007; Grondin, 2010; Allman et al., 2014).

Time and memory are interlinked in that memory provides a mechanism for indexing the passage of time (Olton, 1989). We consider here memory for time itself. Traditional tasks to examine temporal memory are based on single intervals where the listener has to indicate whether the comparison interval is shorter or longer than the reference interval. However, this task based on a binary, categorical response is not well suited to assess influence of memory load and rhythmic context. Temporal generalization (Wearden, 1992) and bisection (Penney et al., 2008) procedures have also been used but these are limited to the retention of one and two interval durations respectively whilst other paradigms have focused on cross-modal effects and memory for temporal order (Grondin, 2005; Gamache and Grondin, 2010).

Influential models of time perception such as the scalar timing models (Gibbon, 1977, 1991) posit that perception of time involves encoding, transfer of duration estimates from working memory to reference memory, and decision making (Gibbon and Church, 1984). Here, the content of temporal memory is determined by the time taken for the clock reading to be transferred from the accumulator to the reference memory. Scalar timing models, however, are better suited to describe temporal processing of single, isolated intervals rather than a sequence of intervals with different rhythmic structure (Church, 1984).

Moving beyond tasks and models based on single intervals is necessary for ecologically relevant analysis of time where multiple time intervals need to be processed for accurate sensorimotor processing such as in the case of speech and music. This requires short-term storage and manipulation of temporal information which brings us to the question of interest in this study: what is the nature of working memory for time intervals?

The nature of working memory itself is currently under debate and there are two major competing theories: the classic account which proposes that working memory is limited in capacity to a set number of items [seven as suggested by Miller (1956); four as proposed by Cowan (2001)] which may be stored in a fixed number of discrete memory slots (Luck and Vogel, 1997) whilst a more recent model proposes that working memory is a limited resource that can be flexibly distributed between all items in a scene (Bays and Husain, 2008; Ma et al., 2014). Resource models do not posit a fixed item limit and emphasize that it is the quality or precision of memory rather than the number of remembered items that defines the limits of working memory (Ma et al., 2014).

In accordance with studies based on resource models of working memory (e.g., Bays and Husain, 2008; Bays et al., 2009; Gorgoraptis et al., 2011; Kumar et al., 2013), we hypothesized that working memory resources for time intervals may also be flexibly allocated between all intervals in a sequence. We quantified temporal memory performance in terms of precision, or the inverse of variance as it provides a continuous index of memory. Precision lends itself nicely to research on time perception as the standard deviation of subject’s estimate is a commonly used index of temporal sensitivity (Grondin, 2001).

In the present study, we designed a novel task that overcomes the limitations of previous paradigms: listeners are required to match the duration of a randomly selected probed interval from a sequence of intervals with variable temporal structure, interval size, working memory load, and attentional cues. In a series of behavioral experiments, we examined precision as a function of the above parameters and demonstrate that working memory resources can be dynamically shared between several temporal “items.”

## MATERIALS AND METHODS

### PARTICIPANTS

All participants in this study reported normal hearing and had no history of audiological or neurological disorders. Experimental procedures were approved by the research ethics committee of University College London, and written informed consent was obtained from each participant. Only participants without extensive (and current) musical training were tested.

Ten listeners (seven females; mean age: 24.6 ± 3.8 years) took part in Experiment 1 (“SUB”) after excluding two listeners because of their inability to perform the task. 10 listeners (seven females; mean age: 23.7 ± 4.5 years) took part in Experiment 2 (“SUPRA”). Eight listeners (six females; mean age: 25.4 ± 6.0 years) participated in Experiment 3 (“MEMORY LOAD”) after excluding two listeners because of poor performance on the task. 10 listeners (four females; mean age: 22.6 ± 4.9 years) participated in Experiment 4A (“CUEING – REG”) whilst another set of 10 listeners (five females; mean age: 21.5 ± 3.2 years) took part in Experiment 4B (“CUEING – IRREG”). The set of participants for all experiments was different in each case.

### STIMULI

The stimulus consisted of a sequence of clicks of 0.5 ms duration and identical loudness. In Experiment 1, the stimulus comprised five clicks that demarcated four time intervals. The inter-onset interval (IOI) was selected from a normal distribution that ranged between 500 and 600 ms. Four different levels of temporal jitter were incorporated: (i) 5–10%, (ii) 20–25%, (iii) 35–40%, and (iv) 50–55%. Higher jitter values increase the difference in duration between the intervals and thus make each interval more unique, resulting in greater memory load. The exact jitter values were randomly drawn from a random distribution between these different ranges of jitter. For each sequence, each IOI was jittered by only one of the above jitter values.

The stimuli for Experiment 2 were identical to that in Experiment 1 except that the IOI ranged from 1000 to 1200 ms. The same four levels of jitter were incorporated.

The stimuli for Experiment 3 consisted of sequences with different number of time intervals, from 1 to 4, and incorporated the same four levels of jitter. The IOI of the sequences in this experiment ranged between 500 and 600 ms as in Experiment 1.

The stimuli for Experiments 4A and 4B consisted of a sequence of four time intervals with an IOI of 500–600 ms that were associated with a jitter of 5–10 and 50–55% respectively.

The stimuli for the control task consisted of a single click only.

### PROCEDURE

Prior to the study, listeners were explained the task and practiced a control reaction time task (12 trials) and the corresponding timing task (16 trials). A single click was presented during the control task and listeners were required to press a button in response. A variable inter-trial interval of 1000–1200 ms separated consecutive trials so that the listeners could not learn to predict the onset of the next click. Listeners were instructed to not respond very quickly during the control task and were instead encouraged to respond at the same rate for both the control and timing trials throughout the duration of the experiment (as responses during the more cognitively demanding timing task would be slower).

In all the timing tasks, a visual probe (e.g., “Match time interval: 1”) was displayed for 1s at the end of each sequence. Here, we used a transient retention phase unlike most other memory experiments where the probed item is presented after a sustained retention phase on the order of several seconds. The probe was presented during a delay period whose duration randomly varied from 800 to 1200 ms. At the end of the delay period, another click was played that represented the start of the interval to be reproduced. Listeners were required to match the duration of the probed interval by pressing a button on a keypad with their right index finger after the onset of this click. A click was presented that coincided with the button press to give the intuitive feeling of having reproduced an interval. Feedback was provided for 500 ms after each trial that indicated the difference between the actual duration of the probed interval and the time matching response (e.g., “Shorter by 27.6 ms” or “Longer by 111.2 ms”). A random inter-trial-interval that ranged between 1.2 and 1.5 s separated successive trials. Each experiment lasted approximately an hour and consisted of 4–5 blocks of 64 timing trials (except for Experiment 3 where a single block consisted of 96 timing trials) that were each preceded by the control reaction time task comprising 40 trials.

In Experiments 4A and 4B, a visual cue was displayed for 2 s before the start of each sound sequence. The cues indicated the interval to be attended for the cued trials (e.g., “ATTEND: 1”). 75% of all trials were cued and 75% of these cued trials were *valid,* i.e., the probe and the cue were the same whilst the remaining trials were *invalid,* i.e., the probe was different from the cue. The remaining 25% of all trials were not cued (marked by the text: “Attend #”) and served as a baseline where listeners were instructed to pay equal attention to all trials, similar to the other experiments.

### ANALYSIS

For the control task, participants’ median reaction time (based on the final 32 of the 40 trials in a block) was computed as the initial responses tend to be more variable. For the timing blocks, the error response was calculated as the difference between the time matching response and the actual duration of the probed interval. The median reaction time was further subtracted from this to obtain a cleaner measure of the perceptual time matching response that was not confounded with the time taken for motor responses.

The absolute value of the error responses was used to calculate precision, or the inverse of the standard deviation (Bays and Husain, 2008). Precision was calculated based on the overall error distribution for each level of the variable of interest respectively: as a function of jitter and serial position of the probed interval (Experiments 1 and 2), the memory load (Experiment 3), and the attentional cue (Experiments 4A and B). Individual precision values were computed for each subject and averaged to obtain the group precision values.

### STATISTICAL ANALYSIS

To test for the main effect of the variable of interest in each experiment: jitter (in Experiments 1 and 2), working memory load (in Experiment 3), and attentional cue (in Experiments 4A and 4B), an analysis of variance (ANOVA) was performed. All statistical tests were conducted in MATLAB R2013b (MathWorks Inc.) using in-built functions from the statistics toolbox. Sphericity was evaluated using the Greenhouse–Geisser correction and the less conservative Huynh–Feldt correction was applied when the epsilon value exceeded 0.70 (Stevens, 1992). Effect sizes (partial eta squares: abbreviated as η^2^) were computed using the Measures of Effect Size toolbox in MATLAB (Hentschke and Stüttgen, 2011). Estimates of effect sizes for *t*-test were evaluated using Rosenthal’s *r* equivalent (Rosenthal and Rubin, 2003).

### APPARATUS

All stimuli were created digitally using MATLAB at a sampling rate of 44.1 kHz and 16 bit resolution. Sounds were delivered diotically through Sennheiser HD555 headphones (Sennheiser, Germany) connected to the output of an external soundcard (Edirol) and presented at a comfortable listening level between 60 and 70 db SPL (self-adjusted by each listener). The stimulus presentation was controlled using Cogent (http://www.vislab.ucl.ac.uk/cogent.php). The participants were tested individually in an acoustically shielded sound booth. The apparatus was identical for all experiments.

## RESULTS

### EXPERIMENT 1: SUB

In Experiment 1, listeners’ memory for the duration of a single sub-second time interval embedded in a sequence of four intervals with different levels of jitter was assessed by computing the precision of the time matching responses.

**FIGURE 1:**
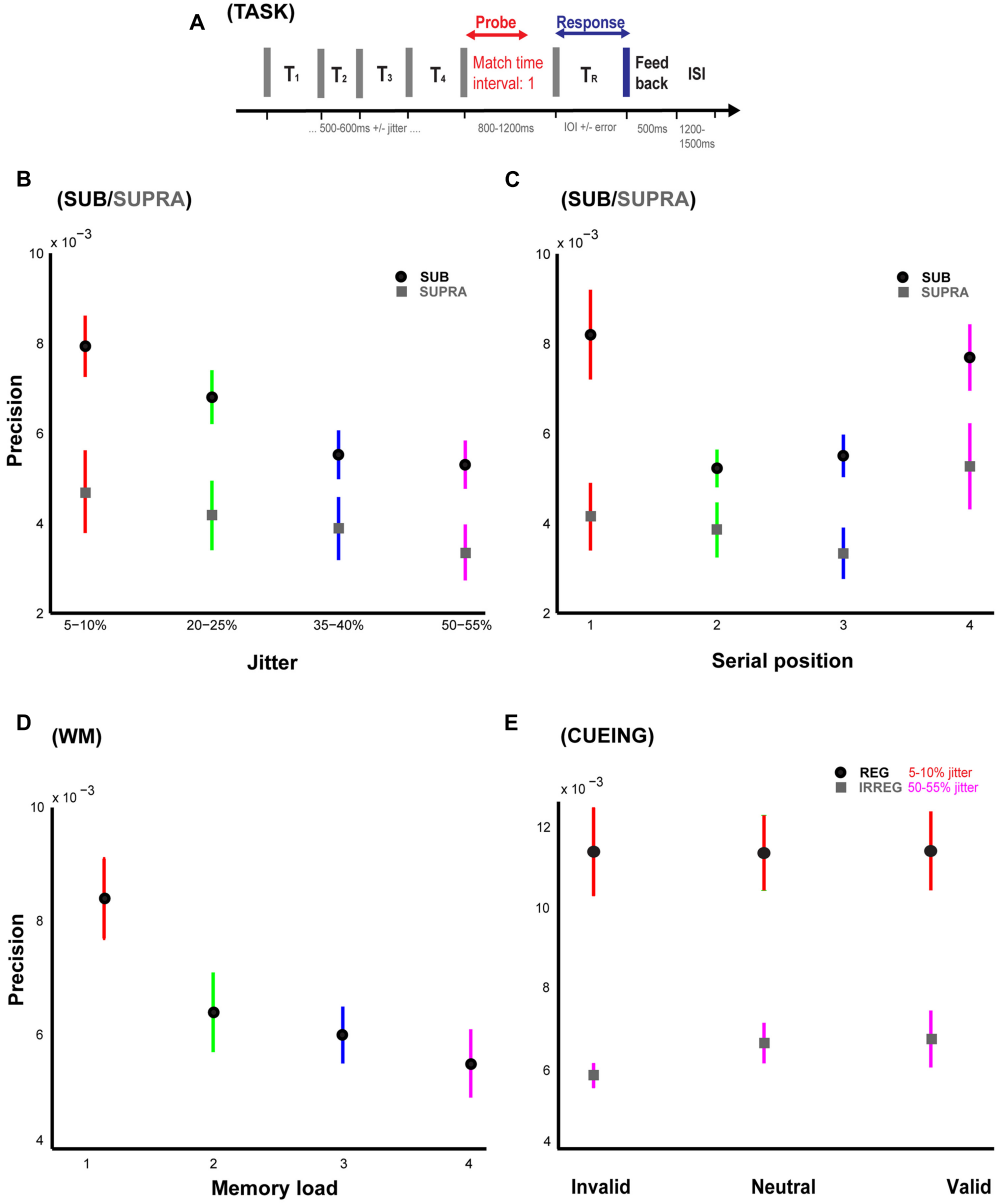
**Temporal memory paradigm and behavioral results. (A)** Stimulus and task. Listeners are presented a sequence of time intervals (four intervals in Experiments 1, 2, 4A and 4B; and 1, 2, 3, or 4 intervals in Experiment 3) separated by clicks. A visual message is used to display the probe interval to be remembered and reproduced at the offset of the last click in the sequence. After a variable delay period, listeners hear another click which signifies the start of the interval to be reproduced by pressing a button when they think that duration equal to the probed interval has elapsed. Feedback or the difference between the duration of the reproduced and the probed interval is presented after each trial. **(B)** Precision vs. temporal regularity in Experiments 1 and 2. Precision or the inverse of standard deviation of the error responses is plotted for the four different levels of temporal jitter [5–10% (red), 20–25% (green), 35–40% (blue), 50–55% (pink)]. Data with the mean indicated by black and gray circles is from Experiments 1 and 2 respectively. **(C)** Precision vs. serial position in Experiments 1 and 2. Precision is plotted as a function of the serial position of the probed interval which could occur randomly at either the first (red), second (green), third (blue), or the fourth (pink) positions. Data with the mean indicated by black and gray circles is from Experiments 1 and 2 respectively. **(D)** Precision vs. working memory load in Experiment 3. Precision is plotted as a function of the number of intervals which was the variable of interest in Experiment 3. The intervals were presented at any of the four jitter levels as in Experiments 1 and 2. **(E)** Precision vs. cue in Experiments 4A and 4B. Precision for invalid, neutral, and valid cues is plotted in blue for a sequence of regular intervals (jitter of 5–10%) and in red for a sequence of irregular intervals (jitter of 50–55%). Error bars represent one SEM.

Analysis of variance was performed to evaluate the effect of the main variable of interest, i.e., jitter on the precision of memory. Results (**Figure 1B**: black circles) indicate a significant effect of jitter (*p* = 0.01, *F*_3,36_ = 4.26, η^2^ = 0.26). Precision decreased with increasing jitter and the percentage drop in precision for the subsequently higher jitter levels compared to the most regular sequence (5–10% jitter) was ∼14% (for 20–25% jitter), 30% (for 35–40% jitter), and 33% (for 50–55% jitter) respectively.

Secondly, precision was also calculated as a function of the serial position of the probed interval (across all jitter values). The results (**Figure 1C**: black circles) indicate a significant effect of serial position (*p* = 0.01, *F*_3,36_ = 4.11, η^2^ = 0.26) as well as the classical primacy and recency effects. There was a significant decrease in precision for position 2 vs. 1 (lower by 36%; *p* = 0.006, *t* = 3.58, df = 9, *r* = 0.77) whilst there was a marginally significant increase in precision for position 4 vs. 3 (higher by 24%; *p* = 0.065, *t* = 2.10, df = 9, *r* = 0.58). There was no significant difference between the precision for the first and last serial positions (*p* = 0.20, *t* = 1.38, df = 9, *r* = 0.42).

An additional analysis was performed to investigate the effect of feedback and whether there was any significant learning across blocks. Feedback was provided to ensure good performance on the task and to avoid random guessing responses. ANOVA results indicate no significant difference in precision (as a function of jitter) across blocks: *p* = 0.10, *F*_4,30_ = 2.16, η^2^ = 0.27 (jitter: 5–10%); *p* = 0.49, *F*_4,30_ = 1.08, η^2^ = 0.15 (jitter: 20–25%); *p* = 0.63, *F*_4,30_ = 0.65, η^2^ = 0.10 (jitter: 35–40%); and *p* = 0.11, *F*_4,30_ = 2.11, η^2^ = 0.26 (jitter: 50–55%).

To obtain an estimate of underestimation or overestimation of responses, the error responses for each jitter level were recomputed using signed instead of absolute values. Results indicate no significant bias across all levels of jitter: *p* = 0.38, *t* = –0.93, df = 9, *r* = 0.30 (jitter: 5–10%); *p* = 0.98, *t* = –0.03, df = 9, *r* = 0.01 (jitter: 20–25%); *p* = 0.91, *t* = 0.12, df = 9, *r* = 0.04 (jitter: 35–40%); and *p* = 0.15, *t* = –1.59, df = 9, *r* = 0.47 (jitter: 50–55%). Positive *t*-values indicate overestimation whilst negative *t*-values indicate underestimation.

### EXPERIMENT 2: SUPRA

The results from Experiment 1 demonstrate that the temporal structure of the sequences significantly affects memory for time intervals in the sub-second range. However, does this sensitivity also extend to sequences containing longer supra-second intervals? This is an important question as there is evidence that perception of time is mediated by different mechanisms and networks for sub- vs. supra-second intervals (Lewis and Miall, 2003; Gooch et al., 2011).

To answer this question, an IOI range of 1000–1200 ms was used in this experiment. The results (**Figure 1B**; gray circles) indicate no significant effect of jitter (*p* = 0.65, *F*_3,36_ = 0.55, η^2^ = 0.04). The decay in precision relative to the most regular jitter level (5–10%) was equal to 11% (for 20–25% jitter), 17% (for 35–40% jitter), and 29% (for 50–55% jitter). These data suggest that memory for time intervals is worse for interval durations longer than a second and does not vary significantly with different levels of temporal regularity unlike the case for sub-second intervals. Furthermore, there was no significant modulation of precision as a function of the serial position of the probed interval (*p* = 0.58, *F*_3,36_ = 0.66, η^2^ = 0.05; **Figure 1C**: gray circles) unlike in Experiment 1.

Similar to Experiment 1, there was no significant learning across blocks: *p* = 0.97, *F*_4,45_ = 0.13, η^2^ = 0.01 (jitter: 5–10%); *p* = 0.99, *F*_4,45_ = 0.07, η^2^ = 0.006 (jitter: 20–25%); *p* = 0.72, *F*_4,45_ = 0.52, η^2^ = 0.045 (jitter: 35–40%); and *p* = 0.72, *F*_4,45_ = 0.52, η^2^ = 0.04 (jitter: 50–55%) and no significant bias across the different jitter conditions: *p* = 0.87, *t* = 0.17, df = 9, *r* = 0.06 (jitter: 5–10%); *p* = 0.90, *t* = –0.12, df = 9, *r* = 0.04 (jitter: 20–25%); *p* = 0.81, *t* = –0.25, df = 9, *r* = 0.08 (jitter: 35–40%); and *p* = 0.93, *t* = –0.09, df = 9, *r* = 0.03 (jitter: 50–55%).

### SCALAR PROPERTY AND TEMPORAL MEMORY

A central pillar of research on interval timing is the scalar property which states that the standard deviation of measures of timing behavior varies linearly with the mean of the time interval (Gibbon, 1977, 1991; Gibbon et al., 1984). This has been observed in human as well as animal timing tasks (Lejeune and Wearden, 2006; Wearden and Lejeune, 2008) and provided another motivation for the second experiment. The range of IOI in Experiment 2 (1000–1200 ms) was exactly doubled from that used in Experiment 1 (500–600 ms) to evaluate whether variance scales linearly and increases by a factor of two (or precision decreases by half) in accordance with the scalar property in our temporal memory task. Previous work suggests that 1.2 s is the limit around which different mechanisms come into play for supra-second compared to sub-second timing (Gibbon et al., 1997; Grondin, 2010) and the choice of IOI (1–1.2 s) in Experiment 2 was made to cover this range. Although the time intervals in each condition were jittered by a different level on each trial, on average the intervals in the second experiment were twice as long as in the first experiment. This is particularly relevant given that no previous experiment has tested the validity of the scalar property as a function of the temporal regularity of sequences or as a function of timing performance on a temporal memory task.

To compare precision in the SUB and SUPRA experiments, an ANOVA with ‘experiment’ as the between-subject factor was performed. Results indicate a significant effect of ‘experiment’: *p* = 0.009, *F*_1,18_ = 8.57. There was no significant interaction between jitter (the within-subject factor) and experiment (the between-subject factor): *p* = 0.26, *F*_3,54_ = 1.36. The ratio of precision values in Experiments 2 vs. 1 were equal to 0.59 (5–10% jitter), 0.61 (20–25% jitter), 0.77 (35–40% jitter), and 0.63 (50–55% jitter) respectively. These data show a trend toward the scalar property and highlight that it may also be observed not only for single interval discrimination tasks but also for intervals embedded in sequences with different temporal structure and for tasks based on temporal memory.

Analysis of precision as a function of serial position between the two experiments revealed a main effect of experiment: *p* = 0.008, *F*_1,18_ = 8.88; a significant effect of serial position: *p* < 0.001, *F*_3,54_ = 7.95 as well as a significant interaction between the two factors: *p* = 0.04, *F*_3,54_ = 2.92.

### EXPERIMENT 3: MEMORY LOAD

The third experiment asked the question whether memory for a single time interval is affected by the number of intervals in the sequence. Here, the stimuli consisted of 1, 2, 3, or 4 time intervals to investigate the effects of working memory load on memory performance.

For each load level, there was no main effect of serial position: *p* = 0.73, *F*_1,14_ = 0.12, η^2^ = 0.009 (load 2), *p* = 0.67, *F*_2,21_ = 0.42, η^2^ = 0.04 (load 3), and *p* = 0.87, *F*_3,28_ = 0.24, η^2^ = 0.02 (load 4). Thus, the average precision was collapsed across all serial positions for each load condition. Results (**Figure 1C**) indicate a main effect of working memory load (collapsed across all jitter values) on precision (*p* = 0.01, *F*_3,28_ = 4.27, η^2^ = 0.31). An interesting aspect of these results is that memory for time continues to decay from 1 to more intervals or “items” in the sequence: the relative drop in precision for successively higher loads with respect to the precision for a single interval was approximately equal to 25, 29, and 35% respectively.

This is contrary to the predictions of classical models of working memory which propose that the brain can hold a fixed number of items in memory (Miller, 1956; Cowan, 2001). Our data suggests that working memory resources for temporal information may be distributed flexibly such that precision of memory is highest for a single item and decreases with more items in the sequence (Ma et al., 2014; see Discussion). The relationship between precision and memory load was fit to a power law (*P*α *N^k^*; adjusted *R*^2^ = 0.96) similar to precision of memory for visual (Bays and Husain, 2008) and auditory features (Kumar et al., 2013) where *N* refers to the number of items. In the case of temporal memory, the rate at which precision decreases as a functional of temporal memory resources, *k* was found to be equal to –0.31 (see Discussion).

In this experiment, there was no significant effect of learning with feedback across the different memory load conditions: *p* = 0.76, *F*_4,34_ = 0.47, η^2^ = 0.05 (load: 1); *p* = 0.91, *F*_4,34_ = 0.25, η^2^ = 0.03 (load: 2); *p* = 0.99, *F*_4,34_ = 0.08, η^2^ = 0.009 (load: 3); and *p* = 0.99, *F*_4,34_ = 0.03, η^2^ = 0.004 (load: 4).

Analysis of the signed error responses revealed significant underestimation in sequences with a single interval: *p* = 0.01, *t* = –3.22, df = 7, *r* = 0.77; whilst there was no significant bias for the other load conditions: *p* = 0.94, *t* = 0.07, df = 7, *r* = 0.03 (load: 2); *p* = 0.57, *t* = 0.60, df = 7, *r* = 0.22 (load: 3); and *p* = 0.47, *t* = 0.76, df = 7, *r* = 0.28 (load: 4).

### EXPERIMENT 4: CUEING

This experiment was motivated by the previous experiment which suggested that working memory resources maybe dynamically allocated as a function of the memory load. Under this assumption, it is possible that memory for a more task relevant item may be preferentially enhanced at the expense of neutral or irrelevant items (Gorgoraptis et al., 2011; Kumar et al., 2013). This hypothesis was tested by cueing time intervals in two different versions of the experiment where listeners were required to attend to specific cued intervals in sequences with either 5–10% jitter (Experiment 4A) or 50–55% jitter (Experiment 4B).

In Experiment 4A, we observed no main effect of attentional cueing, i.e., no significant difference between the precision for valid, invalid or baseline trials (*p* ∼1, *F* ∼0, η^2^ ∼0) although the performance was robust. Analysis of performance across blocks revealed marginally significant learning with feedback for the valid (*p* = 0.056, *F* = 2.50, η^2^ = 0.18) and invalid (*p* = 0.065, *F* = 2.38, η^2^ = 0.17) trials but not for the neutral (*p* = 0.12, *F* = 1.96, η^2^ = 0.15) trials. Lastly, examination of signed error responses revealed significant underestimation of neutral trials (*p* = 0.01, *t* = –3.27, df = 9, *r* = 0.74) with no significant bias for either invalid (*p* = 0.98, *t* = 0.03, df = 9, *r* = 0.01) or valid (*p* = 0.24, *t* = –1.27, df = 9, *r* = 0.39) trials.

Experiment 4B with irregular sequences (50–55% jitter) also revealed no significant effect of attentional cueing (*p* = 0.44, *F*_2,27_ = 0.84, η^2^ = 0.06). The precision in the irregular context was significantly lower than the corresponding values in Experiment 4A with regular sequences: *p* < 0.001, *F*_1,18_ = 26.39, in agreement with the significant effect of jitter on precision (see Experiment 1). Analysis of performance across blocks revealed no significant learning across the different cueing conditions: *p* = 0.54, *F* = 0.72, η^2^ = 0.06 (invalid), *p*= 0.12, *F* = 1.96, η^2^ = 0.08 (neutral), and *p* = 0.36, *F* = 1.11, η^2^ = 0.09 (valid). Investigation of bias revealed an opposite trend compared to the results in Experiment 4A: significant underestimation of responses for invalid (*p* = 0.01, *t* = –3.21, df = 9, *r* = 0.73) and valid (*p* < 0.001, *t* = –4.91, df = 9, *r* = 0.85) trials but not for the neutral (*p* = 0.15, *t* = –1.58, df = 9, *r* = 0.47) trials.

Together, these data demonstrate that increased allocation of attentional resources to task relevant time intervals does not significantly improve time matching performance, contrary to results from similar experiments on attentional cueing of visual (Gorgoraptis et al., 2011) or auditory (Kumar et al., 2013) features (see Discussion).

### EVALUATION OF ALTERNATIVE STRATEGIES

**FIGURE 2:**
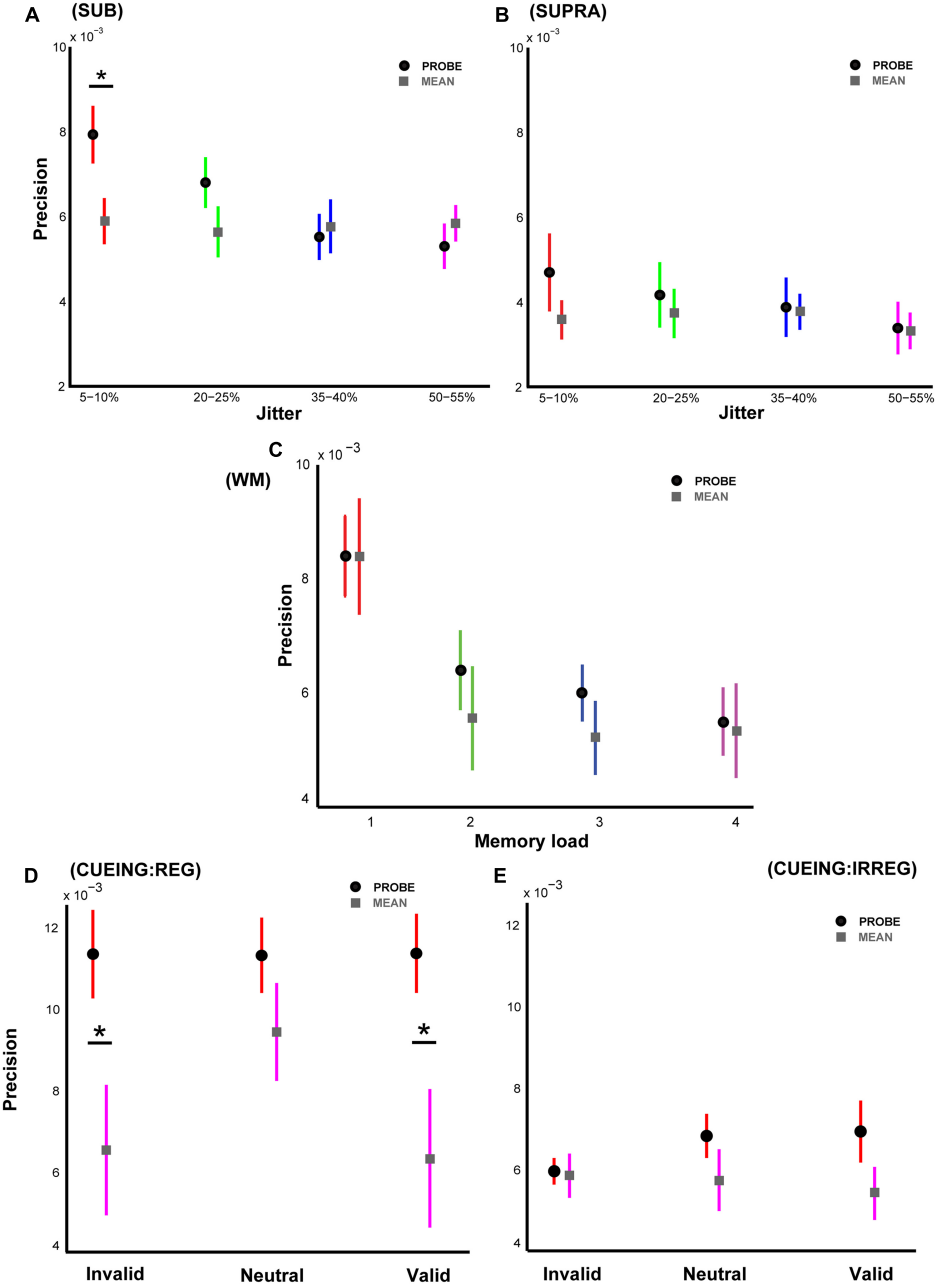
**Experimental results based on responses to average interval duration.** Precision based on a hypothetical response to the average duration of the intervals in a sequence with mean precision in gray squares is plotted alongside the actual precision values with mean precision in black circles as in **Figure 1**. **(A)** Shows the two precision values for Experiment 1, **(B)** for Experiment 2, **(C)** for Experiment 3, **(D)** for Experiment 4A, and **(E)** for Experiment 4B respectively. An asterisk denotes statistically significant difference at a threshold of *p* < 0.05. Error bars represent one SEM.

In this task, listeners were required to match the duration of a single probed interval. However, it is not straightforward to predict listeners’ strategy to recall particular interval durations especially when embedded in sequences with other intervals as well. We investigated the possibility that listeners may simplify their task by computing and responding to the average of the different time intervals in a sequence instead of the specific probed interval. It has been shown previously that the brain can instantiate prior models of temporal uncertainty and adapt its underlying timing mechanisms to temporal statistics in the environment (Jazayeri and Shadlen, 2010; Cicchini et al., 2012). In the time reproduction experiments of Jazayeri and Shadlen (2010), a Bayesian implementation of an ideal observer was demonstrated to be the best explanation of the empirical data. Similarly, in this analysis, we investigated whether listeners were sensitive to the overall temporal statistics of the stimulus and responded to the mean interval instead of the probed interval. For each experiment, hypothetical precision values were calculated by computing the error response as the difference between the time matching response and the mean of all interval durations (instead of the duration of the probed interval). The precision values based on the mean (gray squares) are shown alongside the precision values based on the probed interval (black circles) in **Figure 2**.

**Figure 2A** shows these results for Experiment 1 where there was no difference between the responses to the mean for the different levels of jitter as predicted (as the average duration across jitter levels was held constant, resulting in a fixed average interval duration). A repeated measures ANOVA showed no significant effect of ‘strategy’ (responding to the probe vs. average of all intervals): *p* = 0.86, *F*_1,18_ = 0.03. There was a significant effect of jitter across both response strategies: *p* < 0.001, *F*_3,54_ = 14.42 but no significant interaction: *p* = 0.95, *F*_3,54_ = 0.11. However, there was a significant difference between the responses to the mean and the probed interval for the most regular sequences only: *p* = 0.03, *t* = 2.34, df = 9, *r* = 0.77. Interestingly, with increasing irregularity of the sequences, the difference in precision based on responses to the probe and the mean decreased and showed the opposite trend for the most irregular sequences. Thus, in the face of increasing uncertainty, listeners may improve their task performance by computing summary statistics of the interval distribution (although results indicate that this is not the strategy that listeners actually used). If the listeners performed the task by computing the mean of all the intervals, this would result in similar precision across all serial positions of the probed interval. However, for each experiment we observed significant modulation of precision according to serial position which rules out the ‘mean response’ strategy.

For Experiment 2, a similar pattern of results (**Figure 2B**) was observed although there was no significant difference in precision between the responses based on the probe and the mean: *p* ∼1, *F*_1,18_ ∼0. As expected, the precision based on the mean was similar across the different levels of jitter and smaller than the corresponding precision values in Experiment 1.

Analysis of precision based on the mean for Experiment 3 with variable memory load (**Figure 2C**) revealed no significant differences between the two strategies: *p* = 0.21, *F*_1,14_ = 1.74. There was a significant effect of working memory load (*p* = 0.001, *F*_3,42_ = 6.23) but no significant interaction between response strategy and memory load: *p* = 0.51, *F*_3,42_ = 0.78. However, the data suggest that for sequences with higher memory load (four intervals or more), listeners may benefit from computing the average interval duration.

Results from Experiment 4A (**Figure 2D**) showed a significant difference in precision based on the mean compared to the precision based on the probed interval: *p* = 0.02, *F*_1,18_ = 6.02. There was no significant effect of cueing: *p* = 0.065, *F*_2,36_ = 2.95 but a marginally significant interaction between response strategy and cue type: *p* = 0.057, *F*_2,36_ = 3.11. These data are in agreement with corresponding results from Experiment 1 (for 5–10% jitter condition) and suggest that listeners were actually responding based on the probed interval and taking the average of all intervals does not confer any significant behavioral advantage. The responses based on mean for the neutral trials were lower than the precision based on the probed interval but not significantly different: *p* = 0.11, *t* = 1.77, df = 9, *r* = 0.51.

Similar to Experiment 2 (only the 50–55% jitter condition), Experiment 4B with irregular sequences (50–55% jitter) revealed no significant difference in performance based on the two response strategies (**Figure 2E**): *p* = 0.21, *F*_1,18_ = 1.68.

### CONTROL TASK ANALYSIS

A control analysis was performed to assess whether there was any change in reaction times across the different blocks. This is important to confirm whether listeners’ response times were consistent across blocks and contribute a fixed percentage of (motor) variance that was removed by subtracting the median reaction times (see Materials and Methods).

An ANOVA with session as the between-subject variable showed no significant effect of session for any of the experiments: *p* = 0.99, *F*_4,42_ = 0.05, η^2^ = 0.004 (Experiment 1); *p* = 0.90, *F*_4,45_ = 0.27, η^2^ = 0.02 (Experiment 2); *p* = 0.75, *F* = 0.41, η^2^ = 0.04 (Experiment 3); *p* = 0.28, *F* = 1.31, η^2^ = 0.10 (Experiment 4A); and *p* = 0.92, *F* = 0.16, η^2^ = 0.01 (Experiment 4B).

## DISCUSSION

We designed a novel paradigm to examine memory for time as a function of key temporal and cognitive factors such as temporal structure, interval size, working memory load, and attention. We found that temporal memory decays with the temporal regularity of the sequences. Secondly, time matching performance is better for sub-second vs. supra-second intervals. Thirdly, temporal memory decays with increasing number of intervals in the sequence which supports the predictions of a resource model of working memory where increasing number of items can be encoded into memory but at the cost of decreasing fidelity or precision. Lastly, cueing attention to time intervals in either a regular or irregular sequence did not significantly improve memory performance. We discuss the significance of these results with particular emphasis on implications for models of timing and working memory.

### EFFECT OF TEMPORAL STRUCTURE

Natural sounds consist of temporal patterns that dynamically vary in their levels of temporal regularity. We found that memory for a single time interval depends critically on the temporal structure of the sequences, resulting in better memory for regular vs. irregular sequences. Although previous work has shown that the temporal context of the sequences affects performance on temporal discrimination tasks (e.g., Barnes and Jones, 2000; Teki et al., 2011), these experiments did not specifically examine temporal memory. Instead, they investigated the effect of varying the temporal context of a preceding induction sequence on discrimination of the last two intervals in the sequence. The present paradigm, on the other hand, parametrically probed memory for an interval at any position in the sequence and not only for the second-to-last interval.

Temporal structure influences interval timing and recent evidence has shown the existence of different timing mechanisms: a *duration-based* mechanism is hypothesized to operate for isolated or irregular sequences of intervals and a *beat-based* mechanism for the analysis of time in regular sequences. This well established classification is based on behavioral (Keele et al., 1989; Yee et al., 1994; Pashler, 2001; McAuley and Jones, 2003), neuroimaging (Grahn and Brett, 2007; Grube et al., 2010a; Teki et al., 2011); brain stimulation (Grube et al., 2010b) as well as clinical investigations (Cope et al., 2014a,b). These studies have established the cerebellum (as part of the olivocerebellar network) and the striatum (as part of the striato–thalamo–cortical network) as the core regions mediating duration-based and beat-based perception of time respectively (Teki et al., 2011, 2012; Allman et al., 2014). In the context of memory tasks, these substrates may mediate encoding of time intervals and transfer their content to brain areas hypothesized to serve as temporal accumulators like the insula (Kosillo and Smith, 2010; Wittmann, 2013), the parietal cortex (Leon and Shadlen, 2003), or the hippocampus (Meck et al., 1984).

With reference to models of timing, specifically the scalar timing model, irregular intervals would have highly dissimilar timestamps leading to greater uncertainty at the point of recall. In a regular sequence, on the other hand, there would be less memory mixing and the content of the reference memory would be more similar, resulting in better memory performance (Allman et al., 2014). Models of intrinsic timing based on state-dependent network (SDN) properties of neuronal populations (Goel and Buonomano, 2014) may possibly mediate timing in such complex patterns but it remains to be investigated. Such models propose that short-term plasticity in recurrent neural networks may provide the bases for the memory of an event in the millisecond range (Buonomano, 2000) and present an interesting basis to explore the bases of temporal memory in the context of sequences with variable temporal structure through modeling and neuronal recordings in animal models.

### MEMORY FOR SUB- vs. SUPRA-SECOND INTERVALS

Results from Experiments 1 and 2 suggest differential temporal sensitivity and memory for time intervals in the sub- vs. supra-second ranges. This is in agreement with work suggesting distinct networks for temporal processing in the sub- and supra-second ranges (Lewis and Miall, 2003; Gooch et al., 2011). Timing in the sub-second range is said to be implicit and automatic and mediated by sub-cortical areas including the cerebellum (Koch et al., 2007; Teki et al., 2011) and striatum (Grahn, 2012), whilst supra-second timing requires more explicit encoding and greater cognitive resources in the frontal areas (Lewis and Miall, 2003). Crucially, there was a significant effect of the temporal structure on memory only for the sub-second and not the supra-second intervals that further supports the notion of differential modulation of temporal memory processing as a function of the interval size.

Interestingly, we also observed that the precision of memory in Experiment 2 was approximately half the precision in Experiment 1, in agreement with the scalar property of timing. This suggests that, in the context of the scalar expectancy theory (SET), scalar property is valid not only at the clock stage but also at the memory stage. SDN models, however, do not account for time intervals longer than 500 ms due to the time constants of short-term synaptic plasticity (Karmarkar and Buonomano, 2007).

### EFFECT OF WORKING MEMORY LOAD

Working memory capacity is known to affect temporal discrimination performance in both sub- and supra-second range (Broadway and Engle, 2011). However, these tasks were based on encoding of a single standard interval for later comparison with another interval. Detection of a change in such tasks does not imply perfect recollection of an item; nor does failure to detect imply an absence of memory. To overcome these confounds, we used a continuous response measure and evaluated the quality or precision of memory instead. In Experiment 3 with variable working memory load, we found that precision decays significantly with increasing number of (sub-second) time intervals.

These data suggest that working memory resources for temporal information may be distributed flexibly such that precision is highest for a single item and decreases with more items in the sequence. The data are consistent with a resource model of working memory which has been shown to be valid for a range of visual as well as auditory features (for a review, see Ma et al., 2014), and contrary to models of working memory which assume a fixed capacity (e.g., two or less items for auditory information – Saults and Cowan, 2007; Fougnie and Marois, 2011).

The nature of the relationship between precision and load took the form of a power law (*P*α *N^k^*; *k* = –0.31) in agreement with results from similar paradigms examining memory for pitch (*k* = –0.53, Kumar et al., 2013) and visual orientation/location (*k* = –0.74, Bays and Husain, 2008). These power law relationships suggest that the memory representation of temporal items may be less precise than the representation of auditory or visual items. This loss of fidelity may be attributed either to the serial dependence between successive time intervals, the lack of dedicated temporal processing machinery (in the context of dedicated timing models) or due to the poor temporal selectivity of non-specialized intrinsic neural networks (in the context of SDN models) compared to the selectivity of specialized units for processing sensory information.

In the framework of the SET, the encoded duration of an interval depends upon the time required to transfer the clock reading into reference memory, and the difference between the clock reading and the encoded duration is characterized by a memory translation constant (Gibbon et al., 1984; Meck, 2002; Allman et al., 2014). Extrapolating the SET beyond the context of a single interval, it may be hypothesized that with increasing number of intervals the reference memory gets overloaded, leading to noisier estimates and reduced memory performance, as observed here.

### EFFECT OF ATTENTION

The role of attention in timing has been studied in detail where it has been demonstrated that increased attention to time increases the perceived duration of the interval and results in fewer discrimination errors (Grondin and Macar, 1992; Macar et al., 1994). In one related experiment, listeners were asked to monitor concurrent target stimuli that began or ended at different moments and reproduce the duration of one of these randomly selected stimuli. The results show that the accuracy of timing, as measured by the deviation of a time judgment from a target duration decreases with increasing number of target stimuli, an effect argued to be caused by the allocation of attention to several sources of information (Brown and West, 1990).

However, there has been no previous work on attentional cueing in temporal tasks based on sequences with variable temporal structure. Working memory performance is known to be modulated by task relevance (Awh et al., 2006; McNab and Klingberg, 2008; Kumar et al., 2013), however, results from Experiments 4A and 4B did not show improved memory performance for the cued interval, irrespective of the temporal context. Furthermore, there was no significant overestimation of time intervals as well. It may be possible that the lack of cueing effect may be explained by listeners’ inability to attend to the probed interval (with average duration of 500 ms). The smaller effect size for the regular vs. the irregular version of the experiment also demonstrates the difficulty of attending to an interval in a sequence of highly similar intervals.

The lack of a true behavioral effect suggests that there may be no specific “temporal receptive fields” that show robust tuning for duration and are modulated by attention, like in the case of specialized sensory units for coding visual or auditory features. In terms of models, SET ascribes an attentional switch that gates the pulses from the pacemaker to the accumulator, thereby, increasing the fidelity of temporal representation. However, in the present task, the lack of cueing effects may be attributed to significant interference by other intervals in the sequence. It may be possible that the memory representation of the cued interval is actually enhanced initially but gets degraded in the presence of multiple items in the buffer. Another alternative explanation is that attentional effects would have been observed for longer, supra-second intervals where attentional processes are considered to have a greater influence in comparison to shorter sub-second intervals (as tested here) that rely more on automatic processing (Lewis and Miall, 2003).

## CONCLUSION

Taken together, our data suggests that time intervals may be encoded into memory as objects similar to visual or auditory objects and that the precision of memory depends on several factors including the temporal structure, interval size and the memory load of the sequences. This paradigm based on a resource model of working memory may be extended to model natural scenes (Ma et al., 2014) where several units of temporal information from multiple sources needs to be parsed, for instance, in auditory scene analysis (Teki et al., 2013). Future work will focus on developing a computational framework and investigation of the neural substrates of temporal memory using functional imaging. Additionally, investigation of the oscillatory bases during such temporal memory tasks, for instance, evidence of beta-band modulation by temporal jitter (e.g., Fujioka et al., 2012; Bartolo et al., 2014; Teki, 2014) and alpha-band modulation by number of temporal items will provide novel converging information. Such complementary work will help develop a unified model of context-dependent timing and memory (Teki et al., 2012; Merchant et al., 2013; Allman et al., 2014).

## Conflict of Interest Statement

The authors declare that the research was conducted in the absence of any commercial or financial relationships that could be construed as a potential conflict of interest.
